# Pedicled Rectus Femoris Flap for Restoration of Suprapatellar Quadriceps Tendon and Defect Coverage after Multiple Reconstruction Attempts—A Case Report and Literature Review

**DOI:** 10.3390/jpm14020136

**Published:** 2024-01-24

**Authors:** Andrzej Hecker, Nikolaus Watzinger, Anna-Lisa Pignet, Marlies Schellnegger, Patrick Reinbacher, Werner Girsch

**Affiliations:** 1 Division of Plastic, Aesthetic and Reconstructive Surgery, Department of Surgery, Medical University of Graz, Auenbruggerplatz 29/4, 8036 Graz, Austriawerner.girsch@medunigraz.at (W.G.); 2 COREMED—Center for Regenerative Medicine and Precision Medicine, Joanneum Research Forschungsgesellschaft mbH, Neue Stiftingtalstraße 2, 8010 Graz, Austria; 3 Department of Orthopedics and Trauma, Medical University of Graz, Auenbruggerplatz 5, 8036 Graz, Austria

**Keywords:** pedicled rectus femoris flap, functional reconstruction, suprapatellar knee extensor

## Abstract

There is no unified approach for restoring the suprapatellar quadriceps tendon and covering tissue defects simultaneously. In this case report, we present the pedicled myocutaneous rectus femoris flap as one effective approach in two cases with extensive loss or impairment of the suprapatellar muscle–tendon structures after trauma-related suprapatellar quadriceps tendon rupture and multiple reconstruction attempts. Additionally, we provide a literature review of the reconstructive use of the functional pedicled myocutaneous rectus femoris flap. Methods: Two male patients, 48 and 74 years old, with extensive loss or impairment of the suprapatellar muscle–tendon structures due to multiple reconstruction attempts, underwent restoration of the knee extension with a pedicled myocutaneous rectus femoris flap. Results: Three months after reconstruction, both patients were able to walk freely, unaided. After a six-month follow-up, the free passive mobility of the knee joint was restored, and the active extension of the knee joint was possible in both patients. Conclusion: The authors conclude that the pedicled rectus femoris flap is a reliable method for the restoration of knee extension, with excellent functional results in cases of suprapatellar tendon lesions. Further to the functional restoration, this technique has the additional advantage of simultaneously achieving coverage of soft-tissue defects, while a direct closure of the donor site is possible. Elderly patients and patients with relevant comorbidities or multiple revisions may especially benefit from this technique.

## 1. Introduction

Covering soft tissue defects above the knee presents a challenge to surgeons because a thin and, most importantly, mobile cover is needed to ensure optimal wound healing. Moreover, in some cases, the simultaneous restoration of knee mobility and defect coverage is mandatory, especially in the infrapatellar and suprapatellar flap reconstruction of the knee [1,2]. The need for restoration may arise not only from trauma, post-burn contractures, infections, or the resection of a tumor mass but also from extensor loss after total knee arthroplasty (TKA) [1,3,4].

Flaps to restore knee extension have been used for more than a century. As early as 1897 and 1899, the reconstruction of extension using the sartorius and ischiocrural muscles was described by Goldthwait and Lange [3]. Since then, numerous other techniques have been developed to restore knee extension, especially in infrapatellar tendon rupture. These include using allografts for treating quadriceps deficiency, tendon transfer of the tensor facia lata after femoral nerve palsy, or the utilization of the vastus medialis and lateralis in combination with the soleus muscle and/or medial gastrocnemius muscle through local transfer [5,6]. Another technique makes use of the extended medial gastrocnemius muscle, which was applied as a functional pedicle flap in some cases [7,8]. Here, the additional length offered by the Achilles tendon enables the reconstruction of the suprapatellar extensor mechanism [8]. The medial gastrocnemius muscle flap is more often used for defect coverage around the knee only [1,3]. Donaldson et al. [9] used a pedicled gracilis muscle combined with an adductor longus nerve to regain the quadriceps’ functionality. A different approach to reconstruct knee extensors, especially when the defect cannot be covered with a pedicle flap, are free functional muscular flaps. Concerning these, a free latissimus dorsi or a free contralateral rectus femoris flap has been successfully used for the functional reconstruction of the quadriceps femoris muscle [10,11]. However, free flaps require a longer surgery time and more microsurgical expertise regarding vascular anastomoses and nerve transfers than using a pedicled flap. The rectus femoris muscle has been used as a flap in plastic surgery since 1977 [12]. Since then, this technique has continued to evolve in its application. Due to its versatile properties, it can be used as a local and as a free flap. For instance, it has been utilized for loss of forearm flexors [13], plexus injuries [14], the coverage of defects of the trochanter major in paralyzed patients [15] and for the treatment of infected vascular grafts in the groin [16]. Due to the contractility power of the rectus femoris flap, it has been used as a functional myocutaneous flap to reconstruct traumatic defects of the anterior and posterior compartments in order to restore the dorsal flexion of the foot [17]. As a functional free flap, the myocutaneous rectus femoris flap has also been employed to regain knee extension while simultaneously covering soft tissue defects [11,17]. In addition to the advantage of simultaneous defect coverage, the rectus femoris flap can also be harvested minimally invasively by endoscopy [18].

Due to the wide armamentarium of techniques for reconstructing the knee mechanism, there is no unified approach for restoring the suprapatellar quadriceps tendon and covering tissue defects simultaneously. In this case report, we present the pedicled myocutaneous rectus femoris flap as one effective approach in two cases with extensive loss or impairment of the suprapatellar muscle–tendon structures after trauma-related suprapatellar quadriceps tendon rupture. Additionally, we provide a literature review of the reconstructive use of the functional myocutaneous rectus femoris flap.

## 2. Case Report

### 2.1. Patients

We present two cases with extensive loss or impairment of the suprapatellar muscle–tendon structures around the patella due to multiple reconstruction attempts after trauma-related quadriceps tendon rupture. Before the extensive loss or impairment of the suprapatellar muscle–tendon structures, both patients were mobile without restrictions. Both patients were male and not able to straighten the knee in a sitting position.

#### 2.1.1. Patient 1

**Figure 1:**
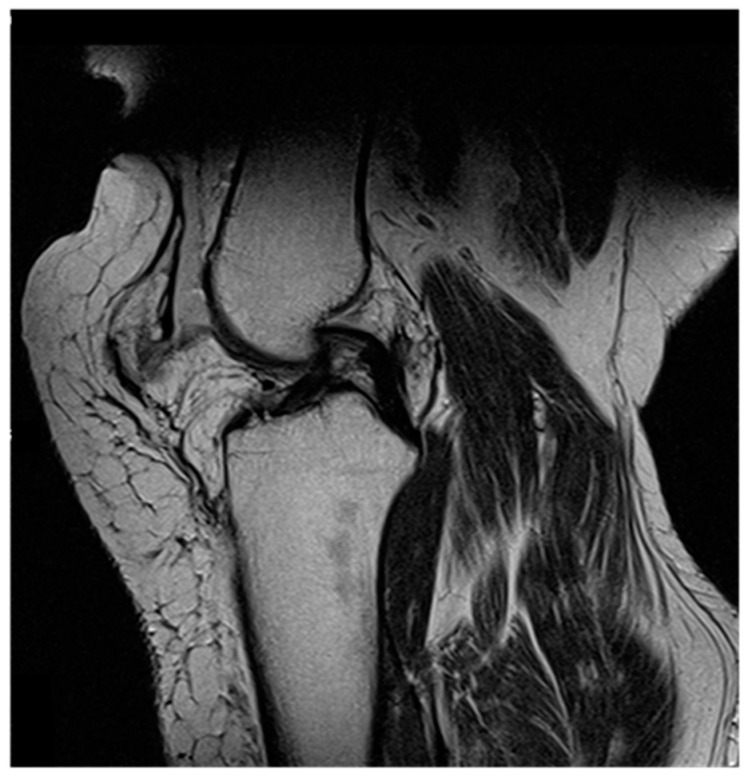
MRI showing overstretching of the knee extensor structures in patient 1.

A 48-year-old male patient with a history of morbid obesity, without any further relevant comorbidities, sustained a rupture of the suprapatellar quadriceps tendon after a minimal trauma, which was reattached with non-resorbable sutures. Shortly thereafter, he suffered a re-rupture of the quadriceps tendon. The tendon was reconstructed with a Ligament Advanced Reinforcement Suprapatellar System (LARS Company, Arc sur Tille, France), an artificial ligament for reconstructing the patella or the cruciate ligament [19]. The patient was remobilized two weeks after surgery, which led, again, to a re-rupture of the quadriceps tendon. From there on, he suffered from functional insufficiency of the involved knee and from pain while climbing stairs. An MRI (magnetic resonance imaging) revealed overstretching of the extensor structures (Figure 1). Through multiple unsuccessful reconstruction attempts, a functional pedicled myocutaneous rectus femoris flap was finally utilized for suprapatellar knee extensor reconstruction.

#### 2.1.2. Patient 2

**Figure 2:**
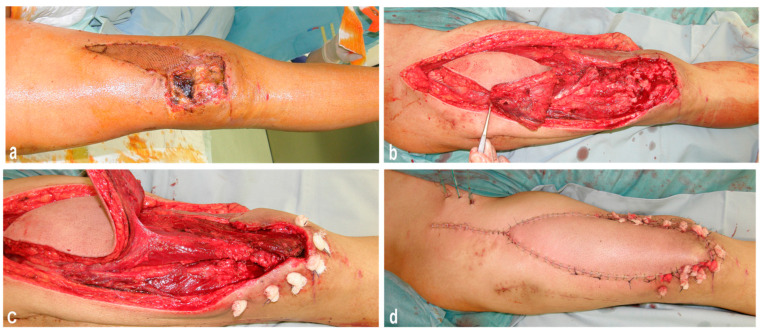
Functional myocutaneous pedicled rectus femoris muscle for the restoration of knee extension and defect coverage after a complex tissue defect caused by multiple reconstruction attempts after a bicondylar femur fracture. (**a**) Complex suprapatellar wound healing disorder after the failure of soft tissue coverage with a local vastus medialis muscle and a split-thickness skin graft. (**b**) After debridement, the patella is exposed down to the patellar tendon and the mycutaneous femoris muscle flap is prepared. (**c**) Intraoperative view of the elevated rectus femoris muscle after dissection from its origin. Here, the rectus femoris flap stays attached only to its vascular pedicle. The rectus femoris muscle is attached distally to the patella with corkscrews. The vastus medialis and lateralis are refixed to the rectus femoris using non-resorbable sutures. (**d**) Finally, the skin island is inserted.

A 74-year-old male patient suffered from a bicondylar femur fracture and complex suprapatellar tissue defect. This patient had no relevant comorbidities such as diabetes mellitus or severe peripheral arterial disease. Before the bicondylar femur fracture, the patient was mobile without any aids. Primarily, the fracture was treated with ORIF (open reduction and internal fixation). For suprapatellar quadriceps tendon coverage, the remnants of the vastus medialis muscle and a split-thickness skin graft were used. Within six weeks, the wound had not healed; therefore, the patient was rescheduled for surgery. The suprapatellar quadriceps tendon and soft tissues were reconstructed at the same time with a pedicled myocutanueos rectus femoris flap (Figure 2).

### 2.2. Pedicled Rectus Femoris Flap—Anatomy and Flap Design

The rectus femoris muscle is a long, thick, fusiform-shaped muscle that is part of the quadriceps muscle and is located in the upper anterior middle compartment of the thigh. It is responsible for hip flexion and knee extension. Moreover, the rectus femoris muscle, as a part of the quadriceps muscle, is one of the major extensors of the knee [20,21]. The muscle originates at the spina iliaca anterior inferior (ASII) and the osseous groove of the acetabulum and inserts into the upper border of the patella along with the vastus lateralis and vastus medialis [20]. The femoral nerve innervates the rectus femoris muscle. Blood supply is provided by the descending branch of the lateral circumflex femoral artery (with a mean diameter of 1.5–2 mm), which is a side branch of the profunda femoris artery [11,20]. The proximal vascular pedicle is usually located 10–15 cm distal to the ASII [22] or 8 cm distal to the inguinal ligament, where it enters the muscle at its deep and lateral surface conjointly with the nerve [23].

### 2.3. Surgical Technique

Existing scar tissue above the knee extensor site was used for the incision by elongating the incision approximately six inches under the ASII. We made sure that the entire anterior surface of the thigh’s skin was exposed in case we needed an additional skin graft for the flap. We performed a radical necrectomy of all soft tissue (Figure 2a). Afterwards, we extended the incision down to the tibial tuberosity to expose the patella and visualize the quadriceps tendon (Figure 2b). The rectus femoris was prepared as a myocutaneous flap, including a skin island over the distal part of the muscle. This muscle was dissected free from the vastus medialis medially, the vastus lateralis laterally, and the vastus intermedius posteriorly (Figure 2c). As a next step, the neurovascular bundle was visualized and released from surrounding tissue to enhance the mobilization of the whole rectus femoris muscle. The muscle was dissected from its insertion of ASII and brought downwards to the recipient area, paying attention not to overstretch the visualized neurovascular bundle. Finally, the distal tendinous part of the rectus femoris was reattached to its original position at the patella using three 3.5 mm corkscrews (Arthrex Inc., Naples, FL, USA). Afterwards, the vastus medialis and lateralis muscles were reattached to the dislocated rectus femoris flap by non-resorbable sutures (Figure 2c). In this way, complete continuity of the suprapatellar muscular extensor apparatus could be achieved. After insertion of drains, the donor site, including the recipient area, was primarily closed (Figure 2d).

### 2.4. Follow-Up Treatment and Clinical Results

At the end of the surgery, a thigh split cast was applied in the extended position of the knee joint for 2 weeks. After the 2 weeks, knee orthoses (Genu Syncro^®^, Mediband, Stockholm, Sweden) was used with a release of the knee joint up to 35° for 2 further weeks. Thereupon, knee orthoses were extended to 45° for two additional weeks, half weight bearing with crutches.

**Figure 3:**
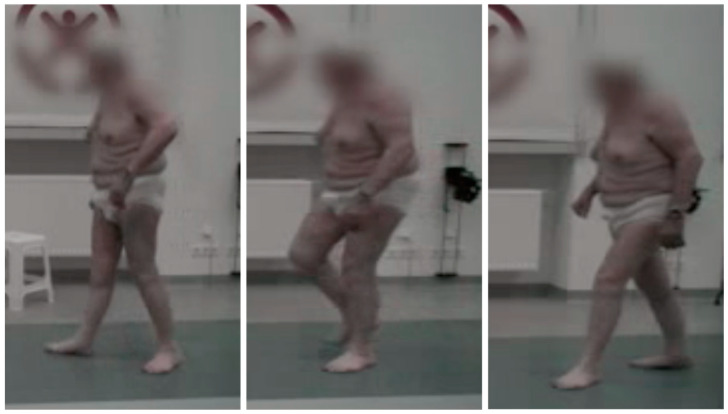
The patient was able to walk again without mobility aids 3 months after suprapatellar knee extensor reconstruction with a pedicled myocutaneous rectus femoris flap.

Starting from the seventh week after surgery, patients were allowed to fully bear weight again. Seven weeks later, walking function improved significantly, and, three months after reconstruction, both patients were able to walk freely without forearm crutches. Wound healing proceeded without complications in both cases. The free passive mobility of the knee joint was restored in each patient during a six-month follow-up after suprapatellar reconstruction with a pedicled myocutaneous rectus femoris flap. The active extension and flexion of the knee joint were possible, except for functionally irrelevant small deficiencies in both patients (Figure 3).

## 3. Discussion

Knee extensor loss is one of the most severe complications after TKA. The loss of infrapatellar knee extensors is reported in 0.17 to 1.4% of TKA cases, and suprapatellar knee extensor loss in 0.11 to 1.1% of such cases, whereas the loss of knee extension after trauma occurs in 0.5 to 6% of cases [3,8,24]. Not only traumatic but also non-traumatic factors, such as diabetes mellitus, obesity, rheumatoid arthritis, and renal insufficiency, increase the likelihood of quadriceps tendon rupture [8]. In addition, unsuccessful surgery or frequent interventions can eventually lead to rupture. Studies have shown that patients underwent an average of three to four knee surgeries before disruption [8]. The causes of the most frequent revisions include infection, pain, loosening of the prosthesis, and deterioration of the TKA [25,26].

Besides TKA-related knee extensor loss, some surgical aspects can negatively impact quadriceps function. For example, an incorrect surgical approach can have a negative effect on the medial-to-lateral balance, resulting in patellofemoral instability and weakness in the knee extension [27]. As already mentioned, knee extensor loss and additional rupture are severe complications, not only after TKA. To restore knee extension after a suprapatellar rupture, suturing the quadriceps to the remaining tendon is one approach. Unfortunately, this technique seems unlikely to produce stable results, and re-rupture happened in one of our patients. The re-rupture of the tendon is very commonly seen and sometimes the induced defect precludes direct closure [8]. Another technique makes use of combined biceps femoris muscle and semitendinosus muscle transfer or a tensor fasciae latae–tendon transfer for regaining knee extension. They are used after femoral nerve palsy, due to poliomyelitis, and extensive tumor resections [6,28]. Nevertheless, these techniques may not apply to simultaneously covering a soft tissue defect. One possible option to restore the knee extension and cover the defect at the same time is to use the extended medial portion of the gastrocnemius muscle. Due to the long vascular portions, good rotation angle, and anatomical proximity to the knee through the additional length offered by the Achilles tendon, this flap can be a good option for reconstructing the suprapatellar extensor mechanism [1,25]. Nevertheless, after utilizing the medial gastrocnemius muscle, the aesthetic results are often unsatisfying, and donor site morbidity can lead to impaired ankle plantar flexion [4]. Utilizing the pedicled myocutaneous rectus femoris muscle as a flap itself preserves functionality and allows suprapatellar tendon reconstruction and soft tissue coverage in a single-stage procedure.

The pedicled myocutaneous rectus femoris flap for functional reconstruction should not be used if the defect exceeds 10 cm in size. Otherwise, there may be a high possibility that the neurovascular bundle is overstretched or may even rupture. Furthermore, the flap cannot be applied if denervation of the femoral nerve has happened. Therefore, as an alternative, free functional muscle flaps should be utilized for the affected area. They are characterized by covering defects of different sizes, good blood supply through anastomoses, and various types of tissues [1,29,30]. Wechselberger et al. [11] already used a contralateral free rectus femoris flap to reconstruct a knee extension with simultaneous defect coverage. Both patients were adolescent and sustained complex trauma with the loss of knee function resulting from extensive traumatic damage to the quadriceps femoris muscle. Here, the free rectus femoris flap led to an excellent functional and aesthetic result in the restoration of knee extension after trauma. Alternatively, a free latissimus dorsi flap can be chosen. They are commonly used for the reconstruction of the knee extension and coverage after removal of tumors and for post-traumatic injuries [10,31]. Unfortunately, the removal of this muscle from its origin can lead to limitations in people who rely on mobility aids such as wheelchairs and crutches [3]. Furthermore, the fusiform shape of the rectus femoris muscle seems to be more suitable for knee extensor reconstruction compared to a strap muscle such as the latissimus dorsi muscle [8]. A free muscle transfer must be deinnerverted and reinnervated after transfer to the recipient site [1]. Additionally, a free muscle transfer diminishes the power of the transferred muscle and needs several months of reinnervation. Especially for elderly populations with comorbidities, these after-transfer aspects can limit reconstructive options.

In the literature, the diverse uses of the functional rectus femoris flap are described, as illustrated in Table 1. This functional flap is employed in various areas, including tumor-related and posttraumatic defects, brachial plexus paralysis, and facial paralysis (Table 1). Even though the use of the myocutaneous rectus femoris flap for functional restoration is described in very few studies, good-to-excellent functional long-term outcomes are observed. Here, the myocutaneous rectus femoris flap was consistently used as a free flap, in contrast to our technique, which utilized a pedicled myocutaneous rectus femoris flap. In some studies, the free myocutaneous rectus femoris flap was also used in combination with other types of flaps (Table 1). The identified studies exclusively comprise case reports or case series, with a correspondingly small cohort and a lack of a control group. These limitations should be considered in the interpretation of the results.

The myocutaneous pedicled rectus femoris flap turns out to be an excellent choice for defect coverage and regaining functionality in the suprapatellar knee extensor apparatus. The ease of harvesting and strong tendon attachment make the rectus femoris flap an appealing approach [11,17]. Due to the direct closure of the donor site, the aesthetic results are convincing. Moreover, it has a better alignment with the surrounding tissue and allows a more natural shaping. Intraoperatively, the suturing of the vastus medialis/lateralis muscle to the rectus femoris has especially proven to be very advantageous in terms of strength [11,17]. However, the relatively short pedicle of this flap limits its application. Elderly patients and patients with relevant comorbidities or multiple revisions may benefit from this technique. Especially after multiple unsuccessful reconstruction attempts, this flap can be considered a good possibility for a functional knee extensor reconstruction. We recommend this technique in cases of extensive loss or impairment of the suprapatellar muscle–tendon structures in combination with an (infection-related) soft-tissue defect. Especially for situations involving infection, we recommend this technique as a first-line therapy. Otherwise, if other techniques are unsuccessful, we recommend the pedicled myocutaneous rectus femoris flap as a second-line option. We do not recommend the use of this technique in cases of severely calcified peripheral arterial disease and the absence or destruction of the patella. Because the vascular pedicle of the rectus femoris flap is too short, thus limiting its application, fixation in the absence or destruction of the patella at the tibial tuberosity would lead to the overstretching of the neurovascular bundle. In conclusion, we are convinced that the pedicled myocutaneous rectus femoris flap is a reliable method for the restoration of knee extension with excellent functional results, which has the additional advantage of simultaneously achieving coverage of the defect.

## 4. Conclusions

The authors conclude that the pedicled myocutaneous rectus femoris flap is a reliable method for the restoration of knee extension with excellent functional results in cases of suprapatellar tendon lesions. Further to the functional restoration, this technique has the additional advantage of simultaneously achieving coverage of soft-tissue defects, while a direct closure of the donor site is possible. Elderly patients and patients with relevant comorbidities or multiple revisions may especially benefit from this technique.

## Figures and Tables

**Table 1:** Applications of functional rectus femoris flap.

Author	Year	Number of Cases	Gender (M:F)	Patient Age (Mean, Range)	Cause of Reconstruction	Location	Flap Type	Follow-Up (Months)
Moschella [32]	2010	1	1:0	72	sarcoma	lower leg	FFRF	18
Wechselberger [33]	2009	1	1:0	22	brachial plexus palsy	upper arm	FFRF	96
Lin [34]	2007	12	7:5	36.8, 22–56	trauma	lower leg	FFRF ^a,b^	3–6
Yang [35]	2006	25	9:16	27, 15–54	facial paralysis	face	FFRF ^c^	15–24
Wechselberger [11]	2006	2	1:1	14.5, 10–19	trauma	thigh	FFRF	27–51
Wechselberger [17]	2004	3	3:0	NA, 17–33	posttraumatic anterior compartment syndrome	lower leg	FFRF	15–39
Koshima [36]	1999	1	0:1	71	abdominal herniation	abdominal wall	FFRF ^d^	54
Koshima [37]	1994	7	NA	NA, 17–59	face paralysis	face	FFRF	4–43
Doi [38]	1993	7	NA	NA	brachial plexus palsy	upper arm	FFRF	NA
Akasaka [39]	1991	17	NA	NA, 16–56	traumatic brachial plexus paralysis	upper arm	FFRF	12
Asko-Seljavaara [40]	1984	1	1:0	20	burns	forearm	FFRF	NA
Schenck [13]	1978	1	1:0	49	trauma	forearm	FFRF	12

M: male; F: female; FFRF: functional free rectus femoris flap; NA: information not available. ^a^ FFRF in combination with an anterolateral thigh flap (*n* = 7); ^b^ FFRF in combination with anterolateral thigh flap, tensor fasciae lata, and iliac crest (*n* = 2); ^c^ FFRF in combination with cross-face nerve graft; ^d^ FFRF in combination with a tensor fascia lata muscle.

## Data Availability

Data are contained within the article.
